# Functional Analysis of General Odorant Binding Protein 2 from the Meadow Moth, *Loxostege sticticalis* L. (Lepidoptera: Pyralidae)

**DOI:** 10.1371/journal.pone.0033589

**Published:** 2012-03-30

**Authors:** Jiao Yin, Honglin Feng, Hongyan Sun, Jinghui Xi, Yazhong Cao, Kebin Li

**Affiliations:** 1 State Key Laboratory for Biology of Plant Diseases and Insect Pests, Institute of Plant Protection, Chinese Academy of Agricultural Sciences, Beijing, People's Republic of China; 2 College of Plant Science, Jilin University, Changchun, Jilin, People's Republic of China; Medical School of Hannover, United States of America

## Abstract

Odorant binding proteins play a crucial role in transporting semiochemicals across the sensillum lymph to olfactory receptors within the insect antennal sensilla. In this study, the general odorant binding protein 2 gene was cloned from the antennae of *Loxostege sticticalis*, using reverse transcription PCR and rapid amplification of cDNA ends. Recombinant *Lsti*GOBP2 was expressed in *Escherichia coli* and purified by Ni ion affinity chromatography. Real-time PCR assays indicated that *Lsti*GOBP2 mRNA is expressed mainly in adult antennae, with expression levels differing with developmental age. Ligand-binding experiments using N-phenyl-naphthylamine (1-NPN) as a fluorescent probe demonstrated that the *Lsti*GOBP2 protein has binding affinity to a broad range of odorants. Most importantly, *trans*-11-tetradecen-1-yl acetate, the pheromone component of *Loxostege sticticalis*, and *trans*-2-hexenal and *cis*-3-hexen-1-ol, the most abundant plant volatiles in essential oils extracted from host plants, had high binding affinities to *Lsti*GOBP2 and elicited strong electrophysiological responses from the antennae of adults.

## Introduction

The meadow moth, *Loxostege sticticalis* (Lepidoptera∶Pyralidae), is one of the worst pests in Asia, Europe, and North America and has caused severe economic damage almost every year [1]. This insect is a polyphagous pest that can feed on 35 plant families and 200 species, but it has an obvious preference for certain host plants [1]–[4]. The meadow moth can find its host using the chemical volatiles emitted by the plant as cues. The plants release volatiles as chemical cues that are diluted and mixed with a myriad of compounds in the environment. With a highly developed olfactory system, the insects can detect low-level signals released from particular plants [5]–[7].

The insects recognize the chemical signals by olfactory receptor neurons in the olfactory sensilla, which are located in the insect antennae and are surrounded by sensillar aqueous lymph that creates a barrier between the external environment and the olfactory receptors, particularly for hydrophobic molecules [8]–[10]. The sensillar lymph contains odorant binding proteins (OBPs), which are believed to carry hydrophobic odorants from the environment to the surface of olfactory receptor neurons [10]–[11]. Odorant binding proteins are the first relay in semiochemical reception in insects, as they enable ligand-receptor interactions. OBPs are water-soluble proteins with a pattern of six conserved cysteine residues. The six conserved cysteines are paired in three disulphide bridges in an interlocking fashion (1–3, 2–5 and 4–6), which are used as a ‘signature’ for identifying insect OBPs [10], [12]–[14]. The first OBP of insects was discovered in the giant moth *Antheraea polyphemus* [8]. Over the last few years, 400 OBPs have been isolated and cloned from more than 40 insect species belonging to eight different orders [15]–[16]. However, even for species for which multiple OBPs have been identified or cloned, the number of binding proteins in the sensillar lymph is significantly lower than the number of compounds the insects can smell. Based on their amino acid sequences, insect OBPs are divided into Pheromone binding proteins (PBPs), general OBPs (GOBPs), and antennal binding protein X (ABPX) [15], [17]–[18]. In many species, the specific binding of proteins to pheromones has been determined for the PBPs, which are located in the pheromone sensilla, the sensilla trichodea [11], [19]–[26]. GOBPs located in the sensilla basiconica are thought to interact with general odorants (e.g., plant volatiles) and are further classified as GOBP1 and GOBP2 [9], [27]–[31]. GOBP2 plays an important role in the detection of general odorants and has a conserved sequence across different species [27]–[28], [32]–[34]. Surprisingly, GOBP2 has been found to have high binding activity to major pheromones in several insect species, some of which are located in the sensilla trichodea [18], [35]–[38].

More than 100 sex pheromones have been used as potential biological agents to control insects through mating disruption or mass trapping strategies [39]. The relationship between plant volatiles and insect behavior is always a hot focal spot for researchers interested in insect control [40]–[44]. In previous works, we demonstrated that the meadow moth has an obvious preference for *Chenopodium glaucum*, and the volatiles of *Chenopodium glaucum* were identified by GC-MS [3] and analyzed the function of a general odorant-binding protein from *Loxostege sticticalis* [45]. The *Lsti*GOBP1 protein has binding affinity to a broad range of test compounds. In the present study, another gene encoding GOBP2 from *Loxostege sticticalis* (*Lsti*GOBP2) was identified, and ligand-binding activities were measured by a fluorescence competitive binding assay using the 1-NPN fluorescent probe. Electroantennograms (EAGs) were used to record the reaction of the antennae to different compounds that can bind to GOBP2 of *Loxostege sticticalis*. The tissue and developmental expression patterns of GOBP2 of *Loxostege sticticalis* were also detected by real-time quantitative PCR (qPCR). In the last, we also compared the binding affinity of two general odorant-binding proteins to discuss the charactering of *Lsti*GOBP2.

## Results

### 1 Coding and amino acid sequences

**Figure 1:**
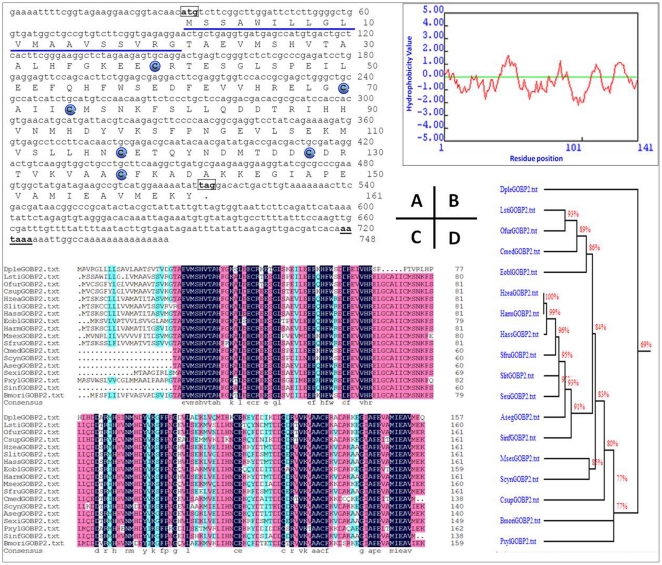
Characterization and Phylogenetic analysis of *Lsti*GOBP2. (**A**) Nucleotide sequence and deduced amino acid sequence of the *LstiGOBP2* cDNA in *L. sticticalis*. The six conserved cysteines are indicated in boxes. The predicted signal peptide is underlined. The asterisk marks the translation-termination codon. (**B**) The predicted hydropathy profiles of the deduced amino acid sequences of the *LstiGOBP2*. The hydropathy profiles was predicted using DNAman 6.3.0.99. (**C**) Alignment of GOBP2 from lepidopteran insects. *Ostrinia furnacalis* (*Ofur*GOBP2, ABG66419)\ *Antheraea pernyi* (*Aper*GOBP2, Q17075)\ *Chilo suppressalis* (*Csup*GOBP2, ACJ07120)\ *Helicoverpa zea* (*Hzea*GOBP2, AAG54078)\ *Helicoverpa assulta* (*Hass*GOBP2, AAQ54909)\ *Spodoptera litura* (*Slit*GOBP2, ABM54824)\*Mamestra brassicae* (*Mbra*GOBP2, AAC05703)\ *Manduca sexta* (*Msex*GOBP2, AAG50015)\ *Heliothis virescens* (*Hvir*GOBP2, Q27288)\ *Ectropis oblique* (*Eobl*GOBP2, ACN29681)\ *Spodoptera frugiperda* (*Sfru*GOBP2, AAT74555)\ *Cnaphalocrocis medinalis* (*Cmed*GOBP2, ACJ07122)\ *Epiphyas postvittana* (*Epos*GOBP2, Q95VP2)\ *Samia cynthia* ricini (*Scyn*GOBP2, BAF91328)\ *Spodoptera exigua* (*Sexi*GOBP2, CAC12831)\ *Agrotis segetum* (*Aseg*GOBP2, ABI24161)\ *Plutella xylostella* (*Pxyl*GOBP2, ABY71035)\*Sesamia inferens* (*Sinf*GOBP2, ACJ07121)\ *Amyelois transitella* (*Atra*GOBP2, ACX47894)\ (**D**) Homology tree of GOBP2 amino acid sequences in Lepidoptera. *Lsti*GOBP2 is indicated in boxes.

Using rapid amplification of cDNA ends (RACE)-PCR, a full-length cDNA encoding GOBP2 was cloned from *L. sticticalis* (Fig. 1A) (GenBank EU239360). The open reading frame (ORF) of *Lsti*GOBP2 cDNA consists of 486 nucleotides and encodes a predicted precursor protein containing 161 amino acids (Fig. 1). The ORF is terminated by a TAG stop codon that is followed by a 232 nucleotide 3′ untranslated region, exclusive of the poly A tail. A consensus polyadenylation signal (AATAAA) is found at 202 bp from the stop codon. The deduced protein sequence revealed a 20-amino acid signal peptide as predicted by SignalP software. The calculated molecular weight and the isoelectric point of mature *Lsti*GOBP2 were 16.0 kDa and 4.76, respectively. *Lsti*GOBP2 had the typical six-cysteine signature of OBPs (Fig. 1A). The hydropathic nature of *Lsti*GOBP2, which is very similar to other insect GOBPs and PBPs [46], was calculated and plotted for each residue in the sequence, revealing that four residue regions were hydrophobic (Fig. 1B).

### 2 Alignment to orthologous of other species

An alignment of the amino acid sequences of *Lsti*GOBP2 with the corresponding GOBP2s from other species of Lepidoptera is shown in Figure 1C. GOBP2 is highly conserved between species. GOBP2s from all Lepidoptera species, including *Lsti*GOBP2, have the typical six-cysteine signature of OBPs and have a common pattern: X_18_−Cys−X_30_−Cys−X_3_−Cys−X_42_−Cys−X_8–10_−Cys−X_8_−Cys−X_24–26_, in which X is any amino acid. *Lsti*GOBP2 shares high identity (70–93%) with other Lepidoptera GOBP2s; the highest identities are 93% with *Ostrinia furnacalis* and 89% with *Cnaphalocrocis medinalis*, which is in accordance with their phylogenetic relationships based on morphological characters (Fig. 1D).

### 3 Expression and purification of recombinant LstiGOBP2

**Figure 2:**
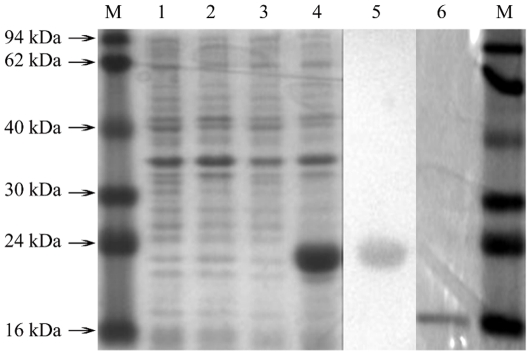
SDS-PAGE electrophoretic and western blot analysis of expressed recombine *Lsti*GOBP2. M. Molecular weight marker; 1. Non-induced *E. coli* pET30; 2. Induced *E. coli* pET30; 3. Non-induced *E. coli Lsti*GOBP2; 4. Induced *E. coli Lsti*GOBP2; 5. Western blot; 6. Purified protein cleaved His by rbEK.

Recombinant *Lsti*GOBP2 was expressed in *E. coli* as a completely soluble protein with high yields (more than 20 mg/L). The His-tag of the recombinant protein was removed by rEK. The protein was purified by two rounds of Ni ion affinity chromatography: the first round was intended to purify the recombinant protein from total protein and the second round was intended to divide the His-tag and the uncleaved His-tagged proteins (Fig. 2). The purified recombinant proteins were then tested for their binding properties and used for the production of polyclonal antibodies.

### 4 Expression pattern analysis of LstiGOBP2

**Figure 3:**
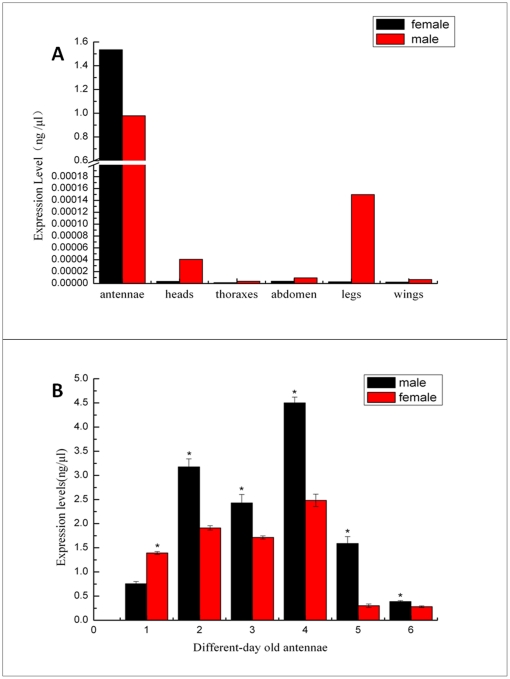
Expression pattern analysis of *Lsti*GOBP2. (A) qPCR analysis of *Loxostege sticticalis* general odorant binding protein 2 (*LstiGOBP2*) expressed in different tissues. cDNAs were amplified with specific primers from antennae, thoraxes, abdomens, wings, legs, tarsite and heads(without antennae). (B) qPCR analysis of *Loxostege sticticalis* general odorant binding protein 2 (*LstiGOBP2*) expressed in different-day old antennae.

We examined the expression pattern of *Lsti*GOBP2 mRNA in different tissues by qPCR. The desired product was largely amplified from cDNA templates that were reverse-transcribed from total RNA in male and female antennae, with only a few derived from other tissues, suggesting that GOBP2 is mainly expressed in antennae (Fig. 3A). In general, the levels of transcripts were very low in all tissues except the antennae, where *Lsti*GOBP2 was highly expressed. However, the expressed quantity of *Lsti*GOBP2 was different in antennae of different ages and there was a notable difference between males and females, with the quantity in male antennae being significantly higher than in female antennae. The quantity of *Lsti*GOBP2 was the highest for both males and females in four-day-old antennae, which is consistent with the age that adults find host plants (Fig. 3B).

### 5 Fluorescence binding assays

**Figure 4:**
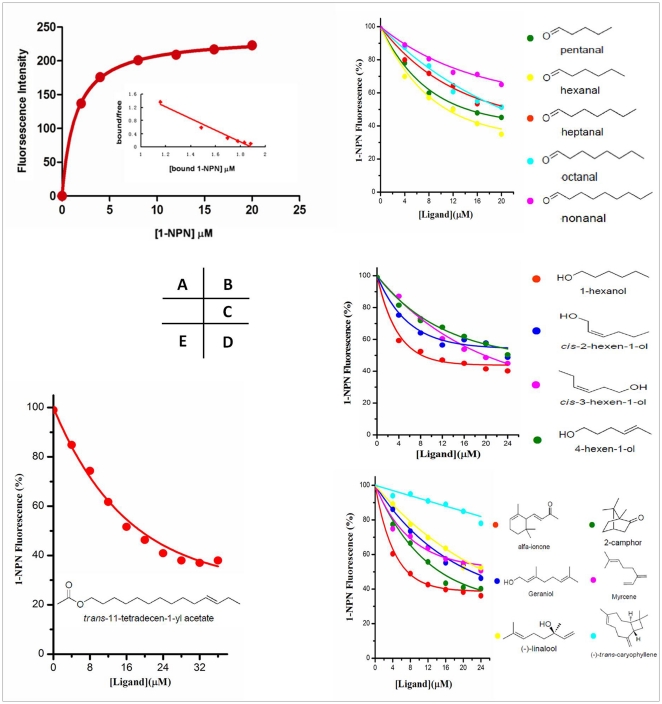
Ligand-binding experiments. (A) Binding curve and relative Scatchard plot. (B) Competitive binding curves of different-carbon-aldehyde ligands to the *Lsti*GOBP2. The chemical structures of the ligands are shown on the right. Mixtures of proteins and 1-NPN, at a 2 µM concentration, were titrated with a 1 mM ligand solution in methanol. (C, D) Competitive binding curves of different structural arrangement ligands to the *Lsti*GOBP2. (E) Competitive binding curves of the sex pheromone component to the *Lsti*GOBP2.

For binding assays with *Lsti*GOBP2, we found that N-phenyl-naphthylamine (1-NPN) was suitable for investigating odorant ligand binding to OBPs. When excited at 337 nm, 1-NPN displayed an emission peak at 380 nm, which shifted to about 420 nm in the presence of *Lsti*GOBP2. The dissociation constant of 1-NPN-bound recombinant *Lsti*GOBP2, approximately 1.44 µM, was calculated according to the changes in fluorescence intensity (Fig. 4A).

Using competitive binding assays, we tested 51 synthetic potential ligands as competitors, including compounds from the leaves of green plants, plant volatiles, high binding affinity ligands of *Lsti*GOBP1 and a sex pheromone component [6], [45], [47]–[48]. Curves for a few representative ligands tested are shown in Figure 4B–4E. Table 1 lists the IC_50_ values (the concentration of ligand halving the initial fluorescence value) and the calculated inhibition constants (*K*_i_) where possible for each OBP/ligand combination. When IC_50_ values of low-affinity ligands could not be calculated, we report the fluorescence intensity (*Int*) measured at the ligand concentration (20 µM) as a percent of the initial fluorescence in the absence of competitor.

**Table 1:** Binding of pure organic compounds to selected recombinant *Lsti*GOBP2.

Ligands	*Lsti*GOBP2			Ligands	*Lsti*GOBP2		
	IC_50_	*Int*	*K_i_*		IC_50_	*Int*	*K_i_*
**Aliphatic alcohols**				**Aromatic compounds**			
1-Hexanol	11	42	6.5	Benzaldehyde	19	48	11.2
Cis-3- hexen-1-ol	19	49	11.2	Cinnamaldehyde	17	45	10
*Cis*-2-hexen-1-ol	24	59	14.1	Phenyl acetaldchyde	20	50	11.8
4-Hexen-1-ol	26	58	15.3	2,4-Di-tert-butylphenol	20	50	11.8
1-Heptanol	10.5	40	6.2	Dimethyl phthalate	36	64	21.2
6-Methyl-5-hepten-2-ol	28	63	16.5	Methyl salicylate	-	83	-
Iso-octanol	28	58	16.5	**Terpenoids**			
**Aliphatic aldehydes**				α-ionone	8	39	4.7
1-Pentanal	14	46	8.2	β-ionone	11	37	6.5
1-Hexanal	12.5	36	7.3	Myrcene	25	56	14.7
*Trans*-2-hexenal	10.5	50	6.2	Camphene	23	54	13..5
1-Heptanal	24	52	14.1	Camphor	14	41	8.2
1-Octanal	24	52	14.1	(-)-Linalool	28	53	16.5
1-Nonanal	39	66	22.9	Geraniol	22	54	12.9
**Aliphatic ketones**				(-)-*Trans-*caryophyllene	-	86	-
6-Methyl-5-hepten-2-one	28	64	16.5	**Heterocyclic compound**			
**Aliphatic ester derivatives**				Benzothiazole	22	52	12.9
*Cis*-3-hexenyl acetate	29	57	17.1	**Sex pheromone component**			
*Trans*-2-hexenyl acetate	28	59	16.5	*Trans*-11-tetradecen-1-yl acetate	18	48	10.6
Dimethyl phthalate	20	50	11.8				

Solution of protein was at 2 µM, and the added concentration of 1-NPN was in line with the dissociation constants of *Lsti*GOBP2/1-NPN complex calculated. Then the mixed solution was titrated with 1 mM solution of each ligand in methanol to final concentrations of 2–50 µM. For the protein, we report the fluorescence intensity (*Int*) measured at the ligand concentration (20 µM) as percent of the initial fluorescence, the concentration of ligand halving the initial fluorescence intensity (IC_50_), where applicable, and the relative dissociation constant (*K_i_*) calculated as described in “Materials and methods”. Dissociation constants of ligands whose IC_50_ exceeded 50 mM are represented as “-”. Other potential ligands were tested, but the remaining 17 potential ligands did not bind *Lsti*GOBP2. These compounds included1-Octen-3-ol, *Trans*-2-hexen-1-ol, R-(+)-Limonene, α-Phellandrene, α-Terpineol, Nerolidol, α-Pinene, Octadecene, Methyl anthranilate, Methyl palmitate, Undecane, Dodecane, Tridecane, Tetradecane, Pentadecane, Hexadecan and Heptadecane.

Most of the volatiles tested succeeded in displacing 1-NPN from the *Lsti*GOBP2/1-NPN complex at concentrations up to 40 mM. The compounds α-ionone, β-ionone, *trans*-2-hexenal, 1-hexanol and 1-heptanol had high binding affinities to *Lsti*GOBP2 with K_i_ values of 4.7, 6.5, 6.2, 6.5 and 6.2 µM, respectively. Interestingly, C11–C17 alkanes did not bind to *Lsti*GOBP2, even though pentadecane has been reported to bind *Lsti*GOBP1 [45]. Most of the aromatic compounds tested in these experiments had a medium binding affinity.

The binding activity of linear aliphatic aldehydes and alcohols to *Lsti*GOBP2 were also tested in our study. We observed that C5–C9 aliphatic aldehydes had different binding activities, and the binding capacities decreased as of the number of carbon atoms increased (Fig. 4B). The binding activities were also different when the compounds differed in structural arrangement, even when the number of carbon atoms remained the same (Fig. 4C, 4D). Surprisingly, trans-11-tetradecen-1-yl acetate, a sex pheromone component, could bind strongly to *Lsti*GOBP2 (Fig. 4E).

### 6 Electroantennogram (EAG) recordings

**Figure 5:**
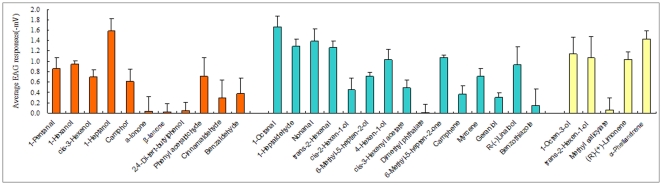
The EAG activity of *L. sticticalis* antennae to different plant volatiles (10 mg/ml). The volatiles in green were the higher competitive binding assay ligand; These in blue showed the moderate affinity and in red could not demonstrate affinity to *Lsti*GOBP2.

**Figure 6:**
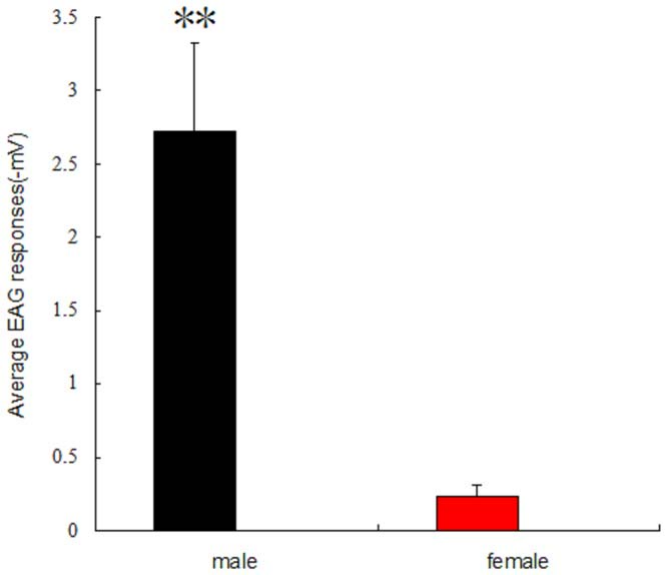
The EAG response difference of *L. sticticalis* antennae to the sex pheromone component between male and female.

Using the results of the competitive binding assays, we selected 31 volatiles to perform EAGs based on their different affinities to *Lsti*GOBP2. The EAG recordings demonstrated that most plant volatiles elicited strong electrophysiological responses from the antennae of female *L. sticticalis* adults at 10 mg/ml (Fig. 5). 1-Heptanol and 1-octanal gave the highest EAG responses and induced a depolarization of −1.6 mV. However, α-ionone, β-ionone and 2,4-di-tert-butylphenol, which were high-affinity competitive binding assay ligands, had only weak effects on both male and female antennae. Conversely, compounds 1-octen-3-ol, E-2-hexeno1, (R)-(+)-limonene and α-phellandrene elicited strong responses from the antennae in spite of their weak affinities to *Lsti*GOBP2. The EAG responses were not statistically different between males and females (data not shown), except in the case of *trans*-11-tetradecen-1-yl acetate. The EAG responses excited by the sex pheromone component were significantly different between males and females (Fig. 6).

## Discussion

Full-length GOBP2 cDNA was cloned from antennae of *L. sticticalis* using RT-PCR and RACE-PCR techniques. The deduced amino acid sequence suggested that the protein should be classified under the insect GOBP2 subfamily, according to the nomenclature proposed by Vogt et al. [9]. *Lsti*GOBP2 shares a high sequence similarity to other insect GOBP2s, especially those of the same family, Pyralidae, *Ostrinia furnacalis* and *Cnaphalocrocis medinalis,* while *Lsti*GOBP1, the other GOBP found in the antennae of *L. sticticalis*, is much less conserved [49]. Based on the complete genome annotation, 61, 72 and 44 OBPs were identified in *Drosophila melanogaster*, the malaria mosquito *Anopheles gambiae* and the silkworm *Bombyx mori*, respectively [46], [50]–[52]. Fourteen OBPs have been identified independently from antennal cDNA libraries of the cotton bollworm, *Helicoverpa armigera* (Hübner) and the lucerne plant bug *Adelphocoris lineolatus* (Goeze) [53], [54]. Therefore, we infer that more than two GOBPs might exist in *L. sticticalis* and diversified functions of these OBPs could be discovered later.

Quantitative examination of transcript levels has revealed that *Lsti*GOBP2 is expressed at a rather high level in the antennae, which implies that *Lsti*GOBP2 is likely to be involved in chemoreception [18], [38]. The transcript levels of *Lsti*GOBP2 are different among male and female moths and also differ between developmental ages. It has been reported that the preoviposition period of female adults lasts for only 4–5 days at 25°C, so having the highest quantity of *Lsti*GOBP2 in 4-day old antennae is beneficial to specific behaviors like oviposition host selection and mating [2]–[3].

Previous research has demonstrated that proteins in the GOBP2 class share high sequence similarity and can bind to a wide range of odors with a broad specificity [18], [27]–[28], [34], [38], [46]. In this study, *Lsti*GOBP2 not only can bind green leaf volatiles, including aliphatic alcohols and aldehydes, but also can bind aromatic compounds and terpenoids. Comparing with the previous study of *Lsti*GOBP1 [45], we found that *Lsti*GOBP2 had a different binding activity compared to *Lsti*GOBP1. In the 50 compounds that we tested, 14 volatiles could displace half 1-NPN from the *Lsti*GOBP2/1-NPN complex at a ligand concentration of 20 µM, but only 4 volatiles could do that from the *Lsti*GOBP1/1-NPN. The binding activity of most of the volatiles tested to *Lsti*GOBP2 was higher than to *Lsti*GOBP1.

Many other researchers have also demonstrated that the length of the carbon chain is critical to the affinities between proteins and ligands [32], [34], [55]–[57]. In our fluorescence competition assays, we found that the binding affinity of these ligands decreased when the number of carbon atoms increased. Both hexanal and pentanal exhibited high affinities to *Lsti*GOBP2, while nonanal had a very weak binding affinity, even at a concentration of 20 µM (Fig. 4B).

Other compounds tested include a collection of different chemicals with the same number of carbon atoms but different functional groups (double bonds, hydroxyl group, aldehyde group, acetyl group or carbonyl group) (Fig. 4C, 4D). Ligands exhibited different binding activities when the position of double bonds was changed in these compounds (Fig. 4C). At the same time, we found that α-ionone and camphor were more competitive than myrcene, geraniol and (-)-linalool (Fig. 4D). There is no obvious difference between these ligands except for the formation of a ring in α-ionone and camphor. Therefore, the spatial structure of ligands can also affect the binding affinity to *Lsti*GOBP2. A ring in the compound perhaps better modulates its interactions with the protein by reducing the number of possible conformations [55]. Thus, the high affinity of 2, 4-di-tert-butylphenol can easily be explained. However, (-)-*trans*-caryophyllene had only a very weak binding affinity to *Lsti*GOBP2, which could be attributed to the size of the ring in (-)-*trans*-caryophyllene, which is too big to enter the binding cavity of *Lsti*GOBP2. The binding activities of α-ionone and β-ionone were significantly different, which indicates that isomeric differences can influence affinity in fluorescence binding experiments. Therefore, *Lsti*GOBP2 might discriminate among odorants on the basis of chain length, functional group, and alkene geometry [58].

Interestingly, we found that the long chain chemical *trans*-11-tetradecen-1-yl acetate, a sex pheromone component of *L. sticticalis* [48], had a high binding affinity to *Lsti*GOBP2. This is consistent with previous studies showing that recombinant *Msex*GOBP2 could be labeled by pheromone analogue (6*E*, 11*Z*)-hexadecadienyl diazoacetate [35], that *Csup*GOBP2 has a high specificity for a major pheromone component 11*Z*-hexadecenal [46] and that *Bmor*GOBP2 can bind to the *B. mori* sex pheromone component (10*E*,12*Z*)-hexadecadien-1-ol (bombykol) [18], [38]. In addition, the native form of GOBP2 from *Mamestra brassicae* (purified from male antennae) did not show affinity to the pheromone components, but displayed a highly specific affinity for *cis*-11-hexadecenol, an antagonist of pheromone-mediated male attraction [59]. Besides, the expression level of *Lsti*GOBP2 in the antennae of male moths was significantly higher than that in antennae of female moths (Fig. 3B) and the EAG response of the male antennae to *trans*-11-tetradecen-1-yl acetate was the highest among all the volatiles tested. Therefore, *Lsti*GOBP2 may also involve in the detection of sex pheromone.

In our study, *Lsti*GOBP2 has been shown to have high binding affinities to *trans*-2-hexenal and *cis*-3- hexen-1-ol, the most abundant plant volatiles in essential oils extracted from the host plant of *L. sticticalis* [3]. During EAG recordings, these chemicals have been found to elicit a high EAG response on their antennae. However, although α-ionone and β-ionone had the strongest affinities for *Lsti*GOBP2, they failed to elicit a strong electrophysiological response on the antennae. Similar results were also reported for 2,4-di-tert-butylphenol, cinnamaldehyde and benzaldehyde, which only exhibited a weak response in spite of their high affinities. It is well known that EAG only represents an overall activity of all the sensilla on the antenna and, therefore, even highly sensitive specialist olfactory receptor neurons may not show up if their total number is low [34]. Conversely, 1-octen-3-ol and *trans*-2-hexen-1-ol induce intensive responses on the antenna, but only present an affinity to *Lsti*GOBP1 [45]. Moreover, antennae have strong responses to volatiles such as (R)-(+)-limonene and α-phellandrene, which do not show affinities to *Lsti*GOBP1 or *Lsti*GOBP2 [45]; similar results have been observed in *Microplitis mediat*or OBPs, *Holotrichia oblita* Fald OBPs and the *Drosophila melanogaster* OBP LUSH [34], [46], [60]–[63].

In fact, many moths have shown a plasticity of olfactory-guided behavior, dependent not only on the nature of the chemical but also on the physiological status (e.g., age/hormone or mating status) of the individual [64]. Laughlin et al. also found that a mutation of OBP causing it to “lock” into a very specific conformation can cause receptor and sensillum activation without the presence of a ligand [26]. The relationship between the transcription of GOBP genes and behavioral plasticity in *L. sticticalis* is very interesting. *Lsti*GOBP2 has been shown to have specialized characteristics, therefore understanding its function is a very important direction in the future. These studies may assist in developing artificial biosensors to monitor odors and devising strategies to disrupt the aggregation behavior of this species.

## Materials and Methods

### 1 Insects and reagents

The meadow moth, *Loxostege sticticalis*, was fed *Chenopodium glaucum* in a laboratory at the Institute of Plant Protection, Chinese Academy of Agricultural Sciences. The antennae, heads (without antennae), thoraxes, abdomens, legs, and wings of the moths were dissected in an insect saline solution containing 0.75% NaCl and stored at −70°C until use.

### 2 RNA extraction, cloning and sequencing

Total RNA was extracted from 20 mg of antennae from *L. sticticalis* females using an RNeasy Mini kit (TIANGEN, China). First-strand cDNA was synthesized using a Prime Script first-strand cDNA synthesis kit (TaKaRa Co., Dalian, China), according to the manufacturer's instructions. Primers were designed by aligning OBP gene sequences from other moths in Lepidoptera.

Based on the sequences of the OBP gene from other moths in Lepidoptera, two degenerate primers, gps4 and gpa4 (gps4: 5′-GTC(G/T)ATGAAA(G)GAC(T)GTCACC(G/T)CTA(C/G/T)GG-3′; gpa4: 5′-AGGTTA(G)AAGTGC(G/T)CG(T)GCTCAT-3′), were synthesized by TaKaRa Company (Dalian, China) for amplification of the partial fragment of *Lsti*GOBP2 cDNA. PCR was performed under the following conditions: three cycles of 40 sec at 94°C, 40 sec at 45°C, and 45 sec at 72°C, and then 30 cycles of 40 sec at 94°C, 40 sec at 47°C, and 45 sec at 72°C. The PCR product was gel purified (TaKaRa Co., Dalian, China), ligated into T-vector (TaKaRa Co., Dalian, China), and the recombinant plasmid DNA was transformed into XL-1 blue competent bacteria. Positive clones were sequenced using the dideoxynucleotide chain termination method (TaKaRa Co., Dalian, China).

Based on the partial sequences of the *Lsti*GOBP2 cDNA, one specific primer, 3R-go1 (5′-GACGGAGGAGTTCTTCCACTTCTG-3′), was synthesized and used with 3 sites adaptor primer (5′-CTGATCTAGAGGTACCGGATCC-3′) for 3′-RACE. Two pairs of nest primers (OP1: 5′-GCTCCAGAAGTGGAAGAACTCCTC-3′; IP1: 5′-CTGTCCGAAGCCGAGGGTGACATCTTT-3′; OP2: 5′-CATGGCTACATGCTGACAGCCTA-3′; IP2: 5′-CGCGGATCCACAGCCTACTGATGATCAGTCGATG-3′) were synthesized for 5′-RACE. 5′- and 3′-RACE were performed following the manufacturer's protocol (SMART™ kit, Clontech). PCR products were sequenced after being inserted into T-vector (TaKaRa Co., Dalian, China).

### 3 Recombinant expression of LstiGOBP2

pGEM plasmid DNAs containing the ORF of *Lsti*GOBP2 were digested with *Noc* I and *Sac* I enzymes for 3 h at 37°C, and the target products were separated on agarose gels. The purified targets were ligated into the expression vector pET30a (Novagen, Germany) and transformed into *E. coli* BL21 (DE3) pLysS cells. A positive clone of *Lsti*GOBP2 in pET30a (pET30a-*Lsti*GOBP2) was identified by PCR and sequencing.

For the expression of recombinant proteins, pET30a-*Lsti*GOBP2 plasmid DNAs were transformed into *E. coli* BL21 (DE3) pLysS cells. After a 3 hr preincubation, recombinant *Lsti*GOBP2 was induced by adding isopropyl-beta-D-thiogalactopyranoside (IPTG) to a final concentration of 0.5 mM for 4 hr. The cells (1L) were harvested by centrifugation, and the pellets were homogenized in phosphate-buffered saline (PBS, 0.04 M, pH 7.0). After centrifugation at 12,000 g for 20 min at 4°C, the supernatants were purified by Ni ion affinity chromatography (GE-Healthcare). To prevent the His-tag from affecting *Lsti*GOBP2 functional studies, the His-tag was removed by recombinant enterokinase (rEK) (Bio Basic Inc.). Uncleaved His-tagged proteins were removed by a second round of Ni ion affinity chromatography. Recombinant *Lsti*GOBP2 was identified with antibodies to 6×His-tag (Abcam, USA) by western blot analysis [65].

### 4 Expression pattern of LstiGOBP2

Total RNA isolated from different tissues was prepared in triplicate. The quality and concentration of the RNA was estimated by determining A260/A280 ratios and then modified to the same (0.1 µg/µl) using DEPC. For RT-PCR, cDNA was synthesized by using a first strand cDNA synthesis kit (TaKaRa Co., Dalian, China). Two primers (GOBP2-FP: 5′-TCCAACAAGTTCTCCCTGCTCC-3′ and GOBP2-RP: 5′-TCCTATCGCAGTCGTCGGTCAT-3′) were used to amplify the cDNA templates of *Lsti*GOBP2. To quantify each mRNA absolute expression level of *Lsti*GOBP2, a credible Standard Curve was constructed using a series 10× diluted standard samples. The PCR reaction conditions were as follows: 95°C for 2 min followed by 42 cycles of 95°C for 15 sec, 61.9°C for 15 sec and 72°C for 30 sec. The expected length of the *Lsti*GOBP2 PCR product was 166 bp. Ct values are presented as mean ± SD for three independent experimental repeats. Real-time qPCR was performed using an iCycleriQ fast real-time PCR system (Bio-Rad, USA), which measures increased fluorescence of the fluorescent dye SYBR (TaKaRa Co., Dalian, China). The Ct value for each tissue and the standard curve were used to calculate the difference of each tissue.

### 5 Fluorescence binding assays

There were 51 compounds used in binding assays, which were purchased from Sigma-Aldrich (Chemie Gmbh, Steinheim, Germany) and had chemical purities >97% (determined by gas chromatography). Fluorescence spectra were recorded in a right angle configuration on a Lengguang 970 CRT spectrofluorimeter (Shanghai Jingmi, China) at room temperature using a 1-cm light path fluorimeter quartz cuvette. Slit widths of 10 nm were selected for both excitation and emission. The spectra data were processed using 970 CRT 2.0l software.

N-phenyl-1-naphthylamine (1-NPN) was dissolved in methanol to yield a 1 mM stock solution. The binding affinity for 1-NPN was determined by adding aliquots of 1-NPN into a 2 µM protein sample to final concentrations of 1 to 20 µM. 1-NPN was excited at 337 nm and emission spectra were recorded between 350 and 600 nm. Spectra were recorded with high-speed scanning. All ligands used in competition experiments were dissolved in spectrophotometric-grade methanol. Binding data were collected in three independent measurements.

Bound ligand was evaluated from the values of fluorescence intensity assuming that the protein was 100% active, with a stoichiometry of 1∶1 (protein∶ligand) at saturation. The K_1-NPN_ values were estimated using Prism 5 (GraphPad Software, Inc.) by nonlinear regression for a unique site of binding. The curves were linearized using Scatchard plots. Dissociation constants of the competitors were calculated according to Campanacci et al. from the corresponding IC_50_ values in the equation: K_i_ = [IC_50_]/1+[1-NPN]/K_1-NPN_, where [1-NPN] is the free concentration of 1-NPN and K_1-NPN_ is the dissociation constant of the complex protein/1-NPN [66].

### 6 Electroantennogram (EAG) recording

Antennae of adult moths were excised at the base and immediately placed on an EAG Micromanipulator MP-15 (Syntech) platform for EAG. Antennae were attached to two electrode holders with non-drying clay (Spectra 360 Electrode Gel). The binding affinity of the compounds to *Lsti*GOBP2 proteins was tested. Pure chemicals were diluted with hexane to a final concentration of 10 mg/ml. EAG signals were amplified, monitored, and analyzed with EAG-Pro software (Syntech). The preparation was held in a humidified air stream delivered by a Syntech stimulus controller (CS-55 model; Syntech) at 500 ml/min, to which a stimulus pulse of 40 ml/min was added for 0.5 min. Signals were recorded for 10 sec, beginning at 2 sec before the onset of the stimulus pulse. An aliquot (10 µl) of each stimulus was loaded onto a filter paper strip. After each sample was tested, hexane was tested as a control. EAG responses of at least three antennae were recorded.
